# Evaluation of Risk Factors for Recurrent Wheezing Episodes

**DOI:** 10.4021/jocmr1543w

**Published:** 2013-08-05

**Authors:** Abdulkadir Bozaykut, Ahu Paketci, Rabia Gonul Sezer, Cem Paketci

**Affiliations:** a Zeynep Kamil Maternity and Childrens’ Disease Training and Research Hospital, Department of Pediatrics, Uskudar 34668, Istanbul, Turkey; b Tekirdag Hospital, Department of Pediatrics, Tekirdag, Turkey; c Namik Kemal University School of Medicine, Department of Pediatrics, Tekirdag, Turkey

**Keywords:** Bronchiolitis, Breastfeeding duration, Infant, Risk factors, Smoking, Wheezing

## Abstract

**Background:**

We aimed to evaluate the risk factors for recurrent wheezing in patients diagnosed with acute bronchiolitis.

**Method:**

From 2009 to 2011, 500 patients from the pediatric clinics, with first attack of acute bronchiolitis were included in this prospective study. Each patient’s age, gender, birth weight, duration of breastfeeding, family history of atopy and asthma, smoking exposure, source of heating in the house, the presence of pets, any history of chronic disease have been questioned. The patients were followed for a duration of 12 - 24 months.

**Results:**

In this study, 39% (n = 195) of the cases were female and 61% (n = 305) were male, with a median age of 3 months old. Male gender, low birth weight (< 2,500 g), low gestational age (< 37 weeks), breastfeeding of less than 6 months, congenital heart disease, family history of atopy, asthma, smoking exposure, stove warming, was found as significant risk factors for recurrent wheezing, however, presence of pets at home was found to be a protective factor.

**Conclusions:**

Informing parents about the risk factors such as exposure to cigarette smoke, heating mode, duration of breastfeeding can significantly decrease recurrent episodes of wheezing.

## Introduction

Acute bronchiolitis is a lower respiratory tract disease characterized by inflammation of the bronchioles. It is often caused by viral agents in children under two years with the symptoms of wheezing, cough, rapid breathing, chest retractions and the incidence is 10-20% [1, 2].

Recurrent wheezing episodes can be seen after acute bronchiolitis attack. Acute bronchiolitis may change the immune response to facilitate the emergence of an acute asthma or acute bronchiolitis in children who already have asthma may result as repeated wheezing attacks. Risk factors for the development of recurrent wheezing episodes are as follows: family history of atopy and allergy in a child, narrow airways, lower socioeconomic status, crowded living conditions, absence of breastfeeding, passive smoking [1, 3]. Pre-existing chronic lung disease, congenital heart disease, immune system dysfunction, premature birth and early infancy are risk factors for serious illness [4]. Children who have school-age siblings, who are in hospital for another reason, those who live in crowded cities, or in day-care centers are at high risk of recurrent attacks [5-7].

Knowing risk factors for acute bronchiolitis and recurrent wheezing attacks is of great importance in terms of child health and development. In this study, we aimed to determine the risk factors for recurrent wheezing in patients diagnosed with first attack of acute bronchiolitis.

## Material and Methods

This hospital-based prospective study included infants with a diagnosis of acute bronchiolitis from the outpatient clinics of Zeynep Kamil Maternity and Children’s Disease Training and Research Hospital between June 2009 and June 2011. The diagnosis of acute bronchiolitis is made on clinical basis; infants with wheezing, retractions, tachypnea, increased respiratory effort with a history of upper respiratory tract infection symptoms such as runny nose, cough, fever were included in the study. Exclusion criterias were as follows: patients with a history of recurrent wheezing, age younger than 1 month and older than 24 months, families who did not agree to participate in the study.

Each patient’s age, gender, gestational age at delivery, birth weight, duration of breastfeeding, family history of atopy and asthma, exposure to cigarette smoke, house warming mode (heating, stove), the number of people living at home, pets at home were recorded for all patients. The presence of chronic disease such as congenital heart disease, immune deficiency was also questioned.

Patients participating in the study were followed up for a period of 12 - 24 months. For each episode of recurrent wheezing, patients were examined by the same doctor and the patients’ and his environments’ characteristics were recorded again. The average duration of each wheezing episode, number of wheezing attacks for each patient during the follow-up period, and the time spent during hospitalization, if any occurred, were recorded. The optimal duration of breastfeeding is defined as 6 months by the World Health Organization, premature birth is defined as birth before 37 weeks of pregnancy, low birth weight is defined as birth weight under 2,500 gr [8, 9]. Recurrent episodes of wheezing is defined as three or more than 3 attacks over the past 12 months [10-14].

The study was approved by the local Ethics Committee of our hospital and an informed consent form was signed by parents who agreed to participate the study.

Statistical Package for Social Sciences, 15.0 was used for the evaluation of data. For the comparison of qualitative data, chi-square test; for the comparison of two groups, Student’s t-test and Mann-Whitney U test; for the comparisons of the average of three groups, Kruskal-Wallis test was used. Logistic and linear regression analysis of the relationship between risk factors was made. Results with a P-value < 0.05 was considered significant.

## Results

A total of 500 infant with acute bronchiolitis were included in this study (Table 1). The median age of patients was 3 (range = 1-24; mean = 6.43 ± 6.21) months.

**Table 1:** Demographic Characteristics of Patients

	n	%
Gender		
female	195	39.0
male	305	61.0
Gestational week		
< 37 week	168	33.6
≥ 37 - 42 week	332	66.4
Birth weight		
< 2,500 g	196	39.2
≥ 2,500 g	304	60.8
Duration of breastfeeding		
≥ 6 months	298	59.6
< 6 months	202	40.4
Congenital Heart Disease		
absent	231	46.2
present	269	53.8
Immune deficiency		
absent	500	100.0
present	0	0.0
Family History of Atopy, Asthma		
absent	432	86.4
present	68	13.6
Exposute to Cigarette Smoking		
absent	267	53.4
present	233	46.6
Pets at Home		
absent	449	89.8
present	51	10.2
House Warming Mode		
stove	325	65.0
central heating	175	35.0

Attack duration of the patients was 4.8 ± 1.6 (median = 5) days, the mean number of episodes was 5.1 ± 2.9 (median = 6). A single episode was seen in 22% of the patients (n = 110), 5.4% (n = 27) had 2 episodes, 72.6% (n = 363) had ≥ 3 (Table 2). Attack duration in patients with ≥ 3 episodes was significantly longer than patients who had <3 attacks (P < 0.05).

**Table 2:** Evaluation of the Number of Wheezing Episodes Based on Risk Factors

Risk factors	Number of Wheezing Episodes		

	Mean	SS	P-value
Gender			
female	3.61	2.27	0.0001
male	6.11	2.94	
Gestational week			
< 37 week	7.48	1.73	0.0001
≥ 37 - 42 week	3.95	2.74	
Birth Weight			
< 2,500 g	6.96	2.17	0.0001
≥ 2,500 g	3.95	2.80	
Duration of breastfeeding			
≥ 6 months	3.75	2.80	0.0001
< 6 months	7.18	1.76	
Congenital Heart Disease			
absent	2.99	2.65	0.0001
present	6.98	1.69	
Family History of Atopy, Asthma			
absent	5.03	2.92	0.320
present	5.76	3.15	
Exposute to Cigarette Smoking			
absent	4.06	2.49	0.0001
present	6.36	2.98	
Pets at Home			
absent	5.50	2.87	0.0001
present	1.94	1.49	
House Warming Mode			
stove	6.42	2.43	0.0001
central heating	2.74	2.28	

Recurrent bronchiolitis (≥ 3 episodes) was significantly higher in male infants, in infants who were born before 37 gestational week and with breastfeeding duration less than 6 months (P = 0.0001) (Table 3).

**Table 3:** Comparison of Patients With One, Two and ≥ Three Wheezing Episodes Based on Risk Factors

	Single Attack		2 Attacks		≥ 3 Attacks		Chi-square	P-value

	n	%	n	%	n	%		
Gender								
female (n: 195)	71	36.4	5	2.6	119	61.0	40.8	0.0001
male (n: 305)	39	12.8	22	7.2	244	80.0		
Gestational week								
< 37 week	4	2.4	2	1.2	162	96.4	72.3	0.0001
38 - 42 week	106	31.9	25	7.5	201	60.5		
Birth weight								
< 2,500 g	11	5.6	2	1.0	183	93.4	69.9	0.0001
≥ 2,500 g	99	32.6	25	8.2	180	59.2		
Duration of breastfeeding								
≥ 6 months	105	35.2	26	8.7	167	56.0	101.6	0.0001
< 6 months	5	2.5	1	0.5	196	97.0		
Congenital Heart Disease								
absent	103	44.6	25	10.8	103	44.6	169.3	0.0001
present	7	2.6	2	0.7	260	96.7		
Family History of Atopy, Asthma								
absent	109	25.2	12	2.8	311	72.0	55.6	0.0001
present	1	1.5	15	22.1	52	76.5		
Exposute to Cigarette Smoking								
absent	83	31.1	12	4.5	172	64.4	27.6	0.0001
present	27	11.6	15	6.4	191	82.0		
Pets at Home								
absent	82	18.3	12	2.7	355	79.1	113.9	0.0001
present	28	54.9	15	29.4	8	15.7		
House Warming Mode								
stove	23	7.1	1	0.3	301	92.6	189.8	0.0001
central heating	87	49.7	26	14.9	62	35.4		

## Discussion

In this study, male gender, low birth weight, low gestational age, breastfeeding of less than 6 months, congenital heart disease, family history of atopy and/or asthma, cigarette smoke exposure, home heating with stove were found to be risk factors for recurrent wheezing.

Male gender is an important risk factor for recurrent wheezing [10-12, 15-17]. In our study, 61% of the patients were male. However, there are studies that could not find any significant difference between males and females [13]. Guilbert et al [18] found that male patients are more sensitive to aeroallergens.

It is shown that duration of breastfeeding should be at least 6 months to be protective against respiratory tract infections [19]. Recurrent episodes of wheezing decreases were with increasing duration of breastfeeding [11, 12, 20-23]. However Taveras et al [24] did not find any difference in terms of recurrent wheezing in children who are breastfed more than 9 months. In this study, recurrent wheezing rate was 54% in infants breastfed less than 6 months. Most of the infants breastfed less than 6 months had additional problems such as prematurity, low birth weight, congenital heart disease and these can also be related to increased number of wheezing episodes. In this study, the number and duration of wheezing episodes decreased as the duration of breastfeeding increased.

In our study, in accordance with the literature, low birth weight and preterm delivery were found to be significant risk factors for recurrent wheezing [11, 16, 22, 24-27]. Recurrent wheezing was present in 44.6% of infants born before 37th gestational week and in 50.4% of infants with low birth weight. Infants with congenital heart disease have significantly more episodes with longer duration at younger ages than infants without congenital heart disease.

Exposure to cigarette smoking is a significant risk factor for recurrent wheezing [11, 20, 26, 28]. In our study, duration of a wheezing attack significantly increased as the passive smoking ratio increased.

Atopy and genetics can be predisposing factors in virus-induced wheezing. It is not clear whether reactive airways are genetically present or occur after acute bronchiolitis [5]. Family history of asthma is a major criteria in asthma predictive index proposed by Coastro-Rodrigez et al [14] and an important risk factor for recurrent wheezing episodes. Reduction of microbial exposure in childhood may be responsible for the increase in allergic diseases [29]. In accordance with the literature [10, 11, 16, 20], in this study, family history of atopy and asthma is a significant risk factor for recurrent wheezing. In our study, infants with family history of atopy had recurrent wheezing with a rate of 91.1%.

Pets at home can be protective against recurrent wheezing episodes [20, 30, 31]. Also in our study, infants with pets at home had significantly lower rates of recurrent wheezing. Only 15.7% of infants with pets at home had recurrent wheezing. The number and duration of episodes was significantly less in infants with pets and the infants’ age at the time of first bronchiolitis was significantly older. Incidence of atopy and asthma was significantly lower in families with pets at home. There are also studies in literature that protective effect of pets was not observed [32, 33]. In our study, presence of pets in the house seemed to be a protective factor for recurrent wheezing, but it is noteworthy that there is no family history of atopy in these families. In the literature there are controversies about pets; some studies have shown that pets are protective, some have shown pets are risk factors and others have shown pets are risk factors if there is atopy in family [13, 16, 20, 22, 26, 27, 30-33]. Therefore, the effect of pet exposure to recurrent wheezing is not clear.

In this study, stove warming was found to be an important risk factor for recurrent wheezing. Most of the infants with recurrent wheezing had stove heating (82.9%) at their homes. Infants whose house heated by a stove had their first bronchiolitis attack at younger ages; higher risk of hospitalization; significantly higher number and duration of attacks.

In our study, patients’ younger age at the time of first acute bronchiolitis can be related to high incidence of premature and low birth-weigh deliveries at our hospital because of the presence of two neonatal intensive care units. Our outpatient clinics have patients characterized as families with low socio-economic conditions and high incidence of complications related to premature birth such as bronchopulmonary dysplasia, congenital heart disease.

It is important to know risk factors for recurrent wheezing so as to predict and prevent recurrent episodes in terms of child health and development. For this reason, patients should be informed about potential risk factors such as tobacco smoke exposure, heating mode, and also the preventive role of breastfeeding. Larger-scaled prospective studies are needed to clarify the effect of pet to recurrent wheezing.
